# AntBot-EX: Enhancing robot search efficiency in complex post-disaster environments

**DOI:** 10.1371/journal.pone.0322980

**Published:** 2025-05-22

**Authors:** Yao Xue, Chee Keong Tan, Wai Peng Wong

**Affiliations:** 1 School of Information Technology, Monash University Malaysia, Jalan Lagoon Selatan, Bandar Sunway, Selangor, Malaysia; 2 Monash Climate-Resilient Infrastructure Research Hub (M-CRInfra), School of Engineering, Monash University Malaysia, Jalan Lagoon Selatan, Bandar Sunway, Selangor, Malaysia; Nazarbayev University, KAZAKHSTAN

## Abstract

In post-disaster scenarios, effective rescue operations hinge on deploying robots equipped with sophisticated path planning algorithms capable of navigating through complex and unknown environments, facilitating an exhaustive search for survivors. The inherent limitations of traditional Coverage Path Planning (CPP) algorithms, particularly their struggle to adapt to the highly dynamic and unpredictable nature of post-disaster environments characterized by collapsed structures, shifting debris fields, and unforeseen obstacles, hinder their effectiveness in time-sensitive rescue operations. To address the challenges, this paper introduces an innovative three-stage online CPP method, termed Ant Colony Optimization based Robot Exploration with Escape Mechanism (AntBot-EX). Our three-stage approach leverages the strengths of different algorithms. Firstly, we utilize a modified Ant Colony Optimization algorithm to explore the unknown environment efficiently, prioritizing uncharted territories and avoiding potential dead ends using an escape mechanism. Secondly, the remaining unexplored areas are segmented, enabling targeted path planning with the ${\text{A}}^{*}$ algorithm to maximize coverage. Thirdly, to address computational limitations in large and complex environments, a configurable boundary-aware and a score-based threshold are introduced to simplify paths by strategically disregarding irrelevant regions, optimizing search efficiency. Simulation results show that our method can basically achieve complete coverage in complex and unknown environments.

## Introduction

Search and Rescue (SAR) operations in the aftermath of natural disasters are a race against time. Collapsed buildings, debris-choked landscapes, and the desperate need to locate survivors trapped within paint a harrowing picture. Traditional search methods, while valiant, struggle in these hazardous environments. Human rescuers face significant risks navigating unstable structures and treacherous terrain. This is where robotics holds immense promise. Robots equipped with advanced navigation and sensor capabilities offer a compelling solution. Despite their ability to access collapsed buildings, traverse rough terrain, and locate survivors in hazardous areas, current robotic SAR technology faces a critical challenge: path planning, especially when integrated with Simultaneous Localization and Mapping (SLAM) and Light Detection and Ranging (LiDAR).

Conventional path planning algorithms for robotic SAR rely on pre-disaster maps. These maps become ineffective in disaster zones due to the dynamic and unpredictable nature of such environments. Earthquakes can cause infrastructure collapse, floods can alter landscapes, and fires can leave behind debris fields. These dramatic transformations render pre-planned paths irrelevant. Consider a scenario where a robot navigates within a building using a pre-defined path. An earthquake could cause a floor to collapse, blocking the planned route. The robot, adhering to its pre-programmed path, would waste valuable time searching for a non-existent passage, potentially delaying critical rescue efforts in other areas. The challenges extend beyond static obstacles. Disaster zones often harbor hidden dangers like exposed electrical wires, unstable structures, and pockets of toxic fumes. Pre-planned paths cannot account for these real-time threats. A robot blindly following a pre-programmed route could be led directly into a hazard zone, jeopardizing its functionality and delaying search efforts. This highlights a critical gap in robotic SAR research. The field requires robust path planning algorithms that function effectively in dynamic and unpredictable environments, seamlessly integrating with SLAM and LiDAR technologies.

This research gap emphasizes the need for online coverage path planning (CPP) for rescue robots. Unlike offline methods, online CPP algorithms leverage real-time sensor data from the robot (e.g., LiDAR, cameras) to navigate and achieve comprehensive exploration of the designated search area. This approach allows robots to adapt their paths dynamically, navigating around unforeseen obstacles and maximizing their search efficiency. However, existing online CPP methods often face limitations when applied to unknown disaster zones. Bio-inspired algorithms (such as ant colony algorithm (ACA), genetic algorithms, particle swarm optimization, etc.) can overcome the limitations of CPP methods to a large extent. First, these algorithms have powerful global search capabilities [1]. Secondly, they are usually processed in parallel and can explore multiple solution spaces at the same time [2]. Finally, these algorithms can dynamically adjust according to environmental changes, have strong adaptability, and are particularly suitable for dynamic and uncertain environments [3]. The popular ant colony algorithm (ACA), for instance, is a bio-inspired approach that mimics the foraging behavior of ants to find optimal paths. While effective in known environments, the ACA can struggle in unknown disaster zones due to several reasons:

- Local Optima and Dead Ends: Unknown obstacles can easily mislead the ACA, causing it to fall into local optima and dead ends. This results in large unexplored areas within the disaster zone.
- Limited Search Space: In environments with many obstacles, ACA emphasizes avoiding repeated paths, thus leading to blind spots and missed search areas.
- Useless and repeated paths: To achieve full coverage search, ACA often requires secondary searches for assistance, but some unimportant or negligible targets often cause a waste of search resources.

This research is motivated by the urgent need to bridge this gap in online CPP for rescue robots [4] [5]. We aim to develop a novel online CPP method specifically designed for the challenges of unknown disaster zones. Despite the above limitations of the ACA, it still shows unique advantages compared to other Bio-inspired algorithms in complex disaster areas, making it a reasonable choice. First, the pheromone mechanism of the ACA makes it more likely that a good path will be selected; at the same time, it ensures the algorithm’s ability to explore new solutions and maintain the diversity of solutions [6]. Second, the ACA can dynamically adjust the search strategy according to environmental changes, showing strong adaptability, especially in dynamic and complex environments [7]. Moreover, the ACA is robust in environmental uncertainty and noise and can work effectively in environments with obstacles, complex terrain, and dynamic change [8]. Therefore, our proposed approach leverages the strengths of the ACA while addressing its limitations in unknown environments. Through three key improvements, we aim to achieve comprehensive and efficient search coverage:

- Addressing Dead Ends and Missed Areas: We incorporate the ant colony optimization based robot exploration with escape in this work to prioritize paths that navigate around obstacles, reducing the likelihood of dead ends. Additionally, a secondary search mechanism specifically targets any remaining unexplored areas after the initial planning phase, ensuring comprehensive coverage of the disaster zone.
- Cellularization and Quadratic Path Planning: By subdividing the unknown environment into smaller cells, the algorithm employs a specific quadratic path planning strategy within each cell. This cellular approach enhances the overall coverage achieved by the ACA and ensures thorough exploration of each subdivided area.
- Full-Coverage Algorithm with Boundary-Aware Limit: To streamline paths and optimize operational efficiency, we introduce a novel "full-coverage with upper limit" algorithm. This algorithm prioritizes exploration based on the size and importance of unvisited cells, strategically focusing on essential regions while expediting the search process.

This paper presents Ant Colony Optimization based Robot Exploration with Escape Mechanism (AntBot-EX), a novel three-stage online CPP method designed for efficient robot exploration in complex and dynamic post-disaster environments. Our approach synergizes the strengths of multiple algorithms to overcome the limitations of traditional CPP methods in such challenging scenarios. In the first stage, a modified ACO algorithm is employed to conduct an initial exploration of the unknown environment. By prioritizing uncharted areas and incorporating an escape mechanism, the robot effectively navigates through the terrain, avoiding potential dead ends. Subsequently, the remaining unexplored regions are segmented to facilitate targeted path planning using the A* algorithm, optimizing coverage within these defined areas. To address computational constraints inherent in large-scale environments, a configurable boundary-aware and score-based thresholding mechanism is introduced, allowing the algorithm to strategically disregard irrelevant regions, thereby enhancing search efficiency.

Our research tackles a critical gap in robotic SAR – the need for robust online CPP. By overcoming limitations in current methods, we’ve developed a CPP approach with the potential to significantly improve SAR effectiveness in disaster zones. This translates to saving more lives during these crucial missions. Through rigorous testing, we’ve shown that our method outperforms existing approaches. It allows robots to navigate uncharted environments efficiently, achieving complete coverage with less exploration time. This paves the way for more successful rescue operations in the complex aftermath of disasters.

## Related works

Complete CPP seeks to identify a path that traverses all points within a designated area or spatial range while circumventing obstacles [9]. In 2000, Choset categorized CPP algorithms into two primary categories based on the availability of a pre-existing environmental map: ‘online’ and ‘offline’ [10]. Offline CPP algorithms leverage solely static environmental information, assuming a priori knowledge of the environment. However, this assumption of comprehensive prior knowledge is often unrealistic in many scenarios. Online CPP algorithms, in contrast, do not necessitate prior knowledge of the target environment. Instead, they utilize sensor data to conduct real-time scans of the space [11]. Consequently, these algorithms are also referred to as sensor-based coverage algorithms. The complete coverage path planning problem addressed in this paper, targeting unknown post-disaster environments, falls within online CPP algorithms.

Currently, based on the different ways of path planning [12], CPP algorithms can be categorized into random methods, cell decomposition methods, template model methods, bio-inspired neural network algorithms, and intelligent algorithms, among others [13–16]. The random collision method involves the robot attempting to cover the work area based on simple movement behaviors [17]. If it encounters an obstacle, it executes a corresponding turn command. The disadvantages of this method are the low efficiency of the coverage algorithm and overly simplistic path planning strategies. The robot often fails to escape dead zones when faced with complex terrains. The cell decomposition method divides the free area of the entire space into simple, non-overlapping subregions called cells. The union of these cells exactly fills the entire free space. The robot covers each subregion using simple coverage patterns (such as back-and-forth or spiral movements). Once each subregion is covered, the entire area is fully covered.

Zhou Linna *et al*. [18] used an ox-plowing algorithm to decompose complex environments into subregions and then applied a bio-inspired neural network algorithm to determine the movement pattern within subregions and path transitions, achieving better coverage in complex environments. However, the algorithm is complex and memory-intensive. Campo *et al*. [19] used a method of local area coverage combined with a backtracking mechanism to revisit and cover any missed areas during the local coverage process, improving the coverage rate but resulting in a high repetition rate. Bähnemann *et al*. [20] used an ox-plowing complete coverage path planning method combined with the cell decomposition methods to handle arbitrary-shaped static obstacles in grid environments. This allowed for quick searches for the next uncovered space, improving efficiency. Nevertheless, it also struggles with coverage in densely scattered obstacle areas. Šelek *et al*. [21] developed a novel algorithm for completely covering known environments inspired by the Spanning Tree Coverage (STC) algorithm. However, the high number of turns reduces operational efficiency. Liu *et al*. [22] used sensor-detected grid map information and a global backtracking mechanism to search for a complete coverage path, improving the robot’s passing ability in densely scattered obstacle areas. However, this approach requires additional sensor information. Le *et al*. [23] designed a complete coverage path planning algorithm based on a spiral spanning tree with a low repetition rate in covering the environment. However, the planned path becomes longer, with more turns, making it prone to dead zones in densely scattered obstacle areas. Wu *et al*. [24] combined genetic algorithms with the ox-plowing cell decomposition method. After dividing the entire free space into sub-regions using partition lines, genetic algorithms were used to encode each sub-region, establish base point information between sub-regions, and obtain the optimal coverage sequence through genetic algorithms. Coverage within each sub-region was achieved through back-and-forth motion, transforming the CPP problem into a Traveling Salesman Problem (TSP). Wang *et al*. [25] introduced the ant colony algorithm into the cell decomposition method. They defined a distance matrix based on the connectivity information between sub-regions and used the ant colony algorithm to optimize the coverage sequence according to the distance matrix. Their experimental results showed that this combined algorithm not only ensures coverage of all workspaces but also results in shorter planned paths, lower path overlap rates, and higher planning efficiency. However, in complex environments, avoiding recovery areas near obstacles is challenging.

Overall, these research achievements have played a positive role in advancing complete coverage path planning technology. However, these algorithms still need help in unknown complex environments, such as high repetition rates, low operational efficiency, and many missed areas. Therefore, this paper proposes a complete coverage path planning algorithm based on the ant colony algorithm for unknown environments. This algorithm has the advantage of simple and feasible operation rules and can address the problem of leaving large unexplored areas when encountering scattered obstacle shapes on an unknown environment map, achieving more straightforward and optimized complete coverage path planning for robots.

## System model and algorithms

### System model

This work addresses the critical challenge of ensuring comprehensive robot coverage within intricate and uncharted post-disaster environments while minimizing traversal distance to conserve battery power. This comprehensive coverage facilitates the subsequent application of SLAM and other techniques to effectively perform mapping and rescue operations. Besides, given the extreme time sensitivity and resource constraints inherent in SAR operations, efficient battery management is paramount for mission success. By optimizing path planning and exploration strategies, this research aims to prolong robot endurance, enhancing the likelihood of locating survivors and mitigating risks to rescue personnel. To achieve this, we propose a novel approach that commences with the creation of a simulated environment to replicate the intricate and unpredictable conditions typical of post-disaster scenarios.

**Fig 1:**
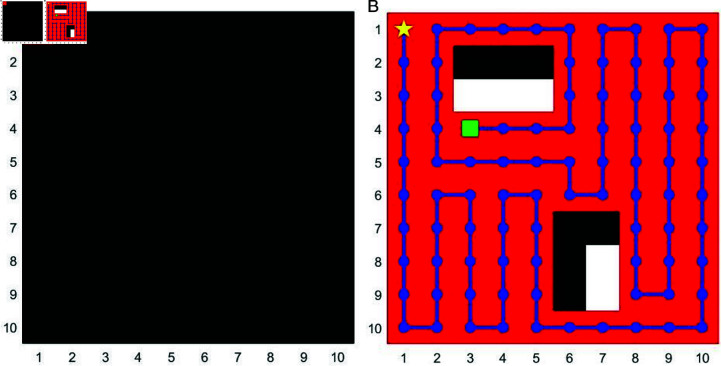
System model. This is the sample of the map creation. (*a*): Unknown map creation. (*b*): The explored map.

We mainly use the gird map built by MATLAB to simulate the unknown environment. In Fig 1(*a*), we establish a grid-based map with each cell measuring 10×10 units. Initially, all cells are colored black to represent the unknown nature of the environment in this initial map. As the robot explores the environment (as shown in Fig 1(*b*)), explored areas are colored red, the robot’s path is depicted in blue, and identified obstacles within explored areas are shown in white. A yellow star marks the starting point, and a green square designates the ending point.

To further enhance the complexity of the simulated environment and make our map closer to the real world terrain, obstacles in the unknown map will be randomly generated and distributed. Each small obstacle will occupy one grid cell, and the total number of obstacles can be adjusted based on the desired level of complexity. We define map complexity as the proportion of the map area occupied by obstacles. According to our criteria, environments with obstacle densities exceeding 10% are considered complex. Since the map and obstacles are randomly generated, we can think that with a high enough complexity, our random map can basically effectively simulate the shape and distribution of obstacles in the real map.

### Ant colony optimization based robot exploration with escape (AntBot-EX)

This work leverages the core concepts of the Ant Colony Algorithm (ACA) by proposing an enhanced variant designed explicitly for exploration within unknown environments. This novel algorithm prioritizes achieving comprehensive coverage (maximizing the explored area) while minimizing the total path length traversed by the robot. This novel algorithm, termed Ant Colony Optimization based Robot Exploration with Escape (AntBot-EX), incorporates an escape mechanism to mitigate the risk of becoming trapped by unforeseen obstacles, operated in a three-stage framework. In the first stage, the decision-making process is augmented by incorporating an additional parameter that quantifies the uncertainty associated with unexplored regions of the map, thereby incentivizing the robot to prioritize the exploration of these areas. To enhance the robot’s adaptability and prevent entrapment by unexpected obstacles, we incorporate an escape mechanism within the core structure of the algorithm.

**Fig 2:**
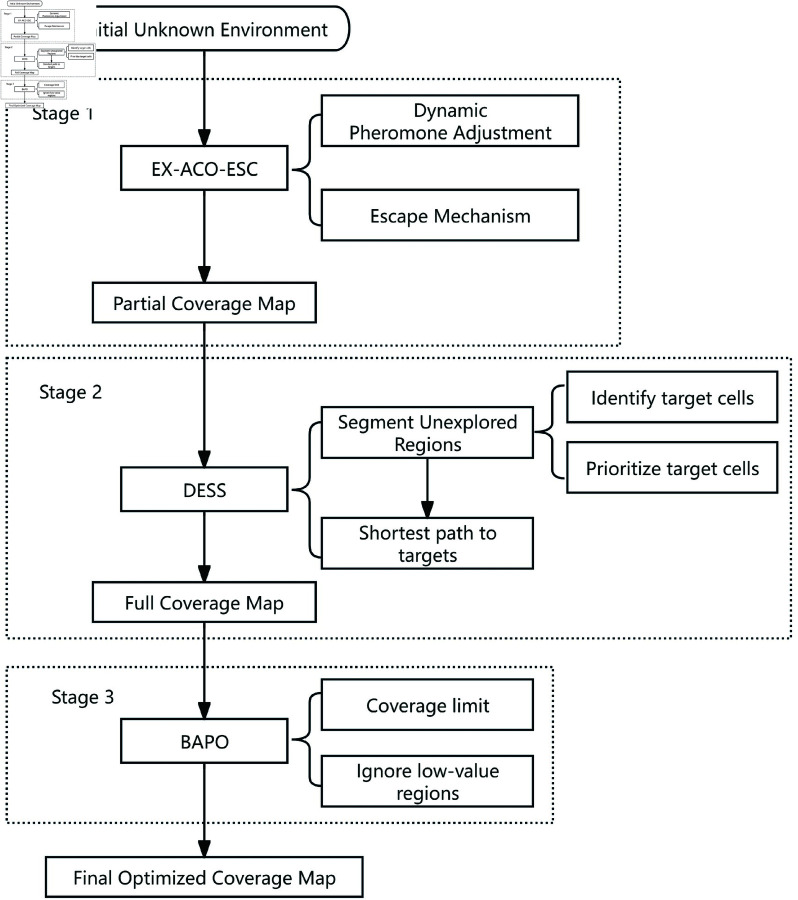
The flowchart of the proposed method.

The entirely unknown nature of the map, coupled with the presence of obstacles, presents a challenge. Certain unexplored regions may receive lower prioritization during the path-planning process, potentially leading to some areas remaining undiscovered within the allotted exploration steps. Therefore, the second stage entails a decision-making process coupled with an algorithm for exploring uncharted regions. At this stage, the unexplored areas are organized into cells, and their priority is determined by evaluating each unexplored unit to explore more valuable unknown areas first. The final stage incorporates a minimum coverage requirements and an boundary-aware limit within the exploration algorithm. Given the inherent uncertainty of obstacles in complex unknown environments, this safeguard aims to mitigate the occurrence of excessive and unproductive search iterations. By restricting the search path length and area, the algorithm improves overall search efficiency and reduces the consumption of computational resources. Fig 2 is the flow chart of our method.

#### Stage 1: Exploratory ant colony optimization with escape mechanism (EX-ACO-ESC).

This first stage of the proposed AntBot-EX leverages a modified Ant Colony Optimization (ACO) algorithm for preliminary exploration within the complex and unknown environment. The goal of this stage is to enable robots to efficiently explore unknown environments while avoiding dead ends. To achieve this, we enhance the classic Ant Colony Optimization (ACO) algorithm with two key mechanisms: 1. Dynamic Pheromone Adjustment: Prioritizes exploration of uncharted regions. 2. Escape Mechanism: Guides robots away from obstacles and dead ends.

To adapt the ACA for effective exploration within unknown environments, we modify the original equation by incorporating several additional factors. These factors serve to enhance the attractiveness of unexplored regions for the virtual ant within the algorithm, thereby increasing their propensity to explore these uncharted territories.

**Fig 3:**
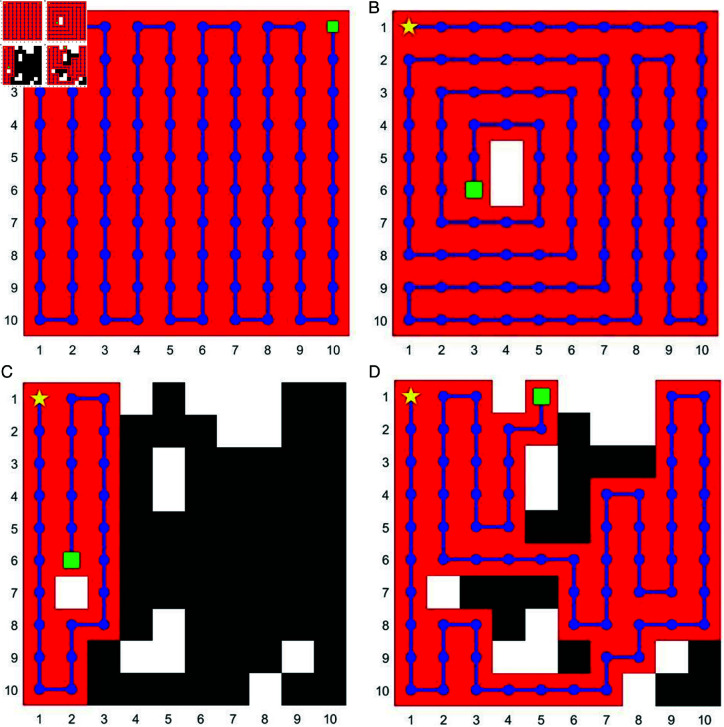
CPP by ACO-CPP and EX-ACO-ESC under different number of obstacles. This is examples of the performance of two methods under different map complexity. (*a*) and (*b*): the performance of ACO-CPP in the environments with absent and minimal obstacles. (*c*): the performance of ACO-CPP in a complex environment (14% complexity). (*d*): the performance of EX-ACO-ESC in a complex environmen (14% complexity)

The first step involves establishing a dynamic adjustment factor for the entire map, denoted by *k*. During the initialization phase, the environment is represented as a grid map, where each cell is initially marked as unknown, while a dynamic adjustment factor *k* is initialized to 1 for all cells, representing the baseline rate of pheromone evaporation. Subsequently, to account for the dynamic exploration challenges, we further adjust the *k* value specifically for unknown areas within the map. For example, in our method, the *k* value of the unexplored area is set to 1.5 to accelerate the evaporation of pheromones in unknown areas. This method prevents excessive repeated visits to the same unexplored area, and promotes the continuous exploration of ant colony. At the same time, this method effectively keeps the pheromone concentration in the unexplored area low. By tailoring the evaporation rate between explored and unexplored regions, we can strategically influence the exploration behavior of the virtual ant within the ACO framework, guiding them toward uncharted territories. (Eq 1) illustrates the calculation of pheromone concentration which can be shown as $\tau_{ij}(t+1)$, depicting pheromone level from position *i* to *j* at time *t* + 1.

$$\tau_{ij}(t+1)\leftarrow (1-\rho \times k_{ij})\times \tau_{ij}(t),$$ (1)

where ρ is evaporation rate of pheromone and *k*_*ij*_ represents the dynamic adjustment factor from position *i* to *j*. The specific value assigned to *k* can be dynamically adapted based on the exploration status of the corresponding location within the map. Within our methodology, pheromone concentration in explored regions directly reflects path quality. It is important to note that the core path selection formula remains faithful to the fundamental ACO formulation. As illustrated in Fig 3(*a*) and 3(*b*), for scenarios with minimal or absent obstacles, the algorithm demonstrates its ability to navigate unknown maps and achieve optimal coverage efficiently.

However, as the number of obstacles increases, the current full-coverage path planning algorithm exhibits a susceptibility to becoming trapped in local optima or dead end in the map. This phenomenon hinders its ability to achieve the desired coverage area, as demonstrated in Fig 3(*c*). To address this limitation, our escape method incorporates a heuristic information design. We introduce *p*_*ij*_ as a penalty parameter based on the distance to obstacles (Eq 3) and $\phi_{ij}$ as a reward term that leverages the attractiveness of unexplored areas (Eq 4). These elements are formalized within the following equations.

$$d(\text{position},o)=|{\text{position}}_{x}-o_{x}|+|{\text{position}}_{y}-o_{y}|,$$ (2)

$$p_{ij}=\sum_{o\in \text{obs}}\frac{1}{d(\text{postion},o)+1},$$ (3)

$$\phi_{ij}=\frac{1}{\sum_{m}{\text{explored}}_{jm}},$$ (4)

where d(position,o) is the distance from current position to obstacle *o*, *p*_*ij*_ is the penalty of obstacles from position *i* to *j*, and $\phi_{ij}$ is the unknown area attraction factor from position *i* to position *j*. Additionally, *m* indicates the index representing the grids around position *j*, and ${\text{explored}}_{jm}$ indicates whether the grid *m* around position *j* has been explored.

Based on the parameter *p*_*ij*_ and $\phi_{ij}$, the path selection probability formula could be further improved as (Eq 5).

$$P_{ij}=\frac{(\tau_{ij}{)}^{\alpha }\cdot (\eta_{ij}{)}^{\beta }}{\sum_{l\in \text{allowed}}(\tau_{il}{)}^{\alpha }\cdot (\eta_{il}{)}^{\beta }},\;\to P_{ij}=\frac{(\tau_{ij}{)}^{\alpha }\cdot (\eta_{ij}{)}^{\beta }\cdot (\phi_{ij}{)}^{\gamma }/(p_{ij}+1{)}^{\lambda }}{\sum_{l\in \text{allowed}}(\tau_{il}{)}^{\alpha }\cdot (\eta_{il}{)}^{\beta }\cdot (\phi_{il}{)}^{\gamma }/(p_{il}+1{)}^{\lambda }},$$ (5)

We can further simplify the formula as,

$${\text{score}}_{ij}=\frac{(\tau_{ij}{)}^{\alpha }\cdot (\eta_{ij}{)}^{\beta }\cdot (\phi_{ij}{)}^{\gamma }}{(p_{ij}+1{)}^{\lambda }},$$ (6)

$$P_{ij}=\frac{{\text{score}}_{ij}}{\sum_{l\in \text{allowed}}{\text{score}}_{il}},$$ (7)

where ${\text{score}}_{ij}$ is the score from position *i* to position *j*. Besides, α is the weight of the pheromone concentration, β is the weight of heuristic information to control the length of the movement path, ϕ is the weight of the attractiveness of the unknown region, and λ is the weight of the obstacle penalty. Moreover, ‘ allowed ’ is the set of next positions that ants are allowed to choose.

The algorithm uses the probability formula to guide the ants towards paths with higher heuristic information. In essence, paths situated further away from obstacles hold a greater probability of selection. At the same time, paths will also be directed towards directions with more unknown areas. By promoting the selection of these paths, the ants exhibit a reduced tendency to become trapped in local optima, as illustrated in Fig 3(*d*). The pseudo-code for this specific component is presented in Algorithm 1.

**Algorithm 1. Exploratory ant colony optimization with escape mechanism (EX-ACO-ESC).**

1: Initialize the score set scores and the position set

positions

2: **for** each direction dir **do**

3:   Calculate the new position newPos, formula: newPos =

currentPos + dir

4:   **if** the new position is within map bounds, not visited,

   and not an obstacle **then**

5:    Calculate the punish parameter

6:    Calculate the appeal of unexplored areas


$$\phi_{ij}=\frac{1}{\sum_{m}{\text{explored}}_{jm}}$$

7:    Calculate the score


$${\text{score}}_{ij}=\frac{(\tau_{ij}{)}^{\alpha }(\eta_{ij}{)}^{\beta }(\phi_{ij}{)}^{\gamma }}{(p_{ij}+1{)}^{\lambda }}$$

8:    Add the score and position to the sets scores and

   positions

9:   **end if**

10: **end for**

11: **if** scores set is not empty **then**

12:   Calculate selection probabilities


$$P_{ij}=\frac{{\text{score}}_{ij}}{\sum_{l\in \text{allowed}}{\text{score}}_{il}}$$

13:   Choose the next position nextPos based on probabilities

14: **else**

15:   nextPos = [ ]

16: **end if**

#### Stage 2: Directed exploration and secondary search (DESS).

**Fig 4:**
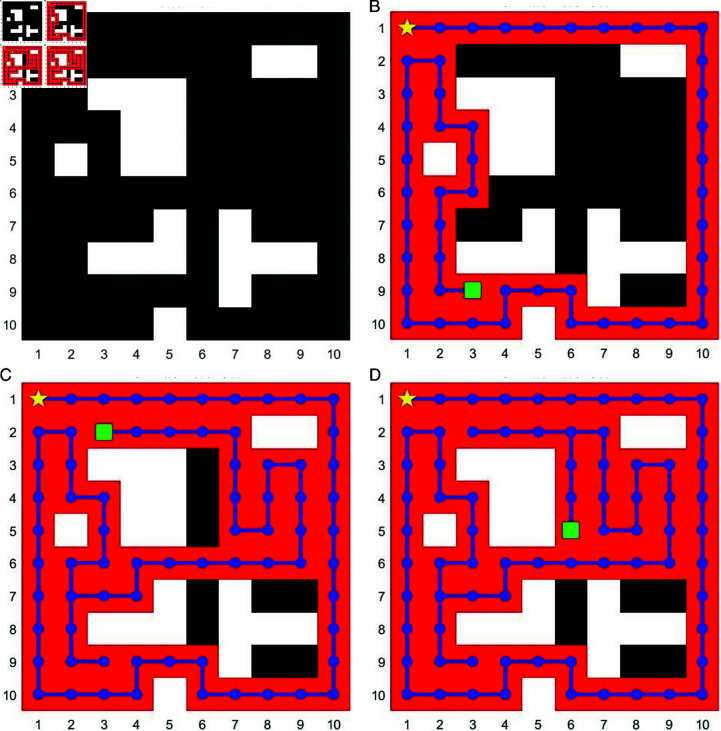
CPP performance achieved by incorporating DESS into EX-ACO-ESC. This is examples of the DESS’s performance. (*a*): the original unknown map with 20% complexity. (*b*): the path planning by EX-ACO-ESC. (*c*) and (*d*): the path planning by both EX-ACO-ESC and DESS for the same map with different ending points.

As shown in Fig 3(*b*), in simpler environments with fewer obstacles, especially those clustered at the edges, the coverage of the first stage of AntBot-EX can reach impressive levels above 95%. However, as shown in Fig 3(*d*), despite improving coverage, the algorithm’s inherent bias towards shortest path optimization often compromises comprehensive area exploration, particularly in complex environments. As environmental complexity increases, this tendency becomes more pronounced, hindering the achievement of optimal coverage. If the complexity increases for the environment as illustrated in Fig 4(*a*), this behavior leads to a significant drop in coverage rate as obstacles increase, hindering its effectiveness in exploring intricate post-disaster environments as shown in Fig 4(*b*). To address this limitation, we propose a secondary search method to address coverage gaps left by Stage 1, especially in high-obstacle density areas. This part focuses on two key aspects: evaluating target locations and finding the shortest paths to reach them.

Fig 4(*b*) shows the initial unexplored map, with a complexity of 20%. After completing the first stage of route planning using Algorithm 1, the path is shown in Fig 4(*b*), where we can see poor coverage due to the obstacles. To tackle this issue, we use the explored areas and obstacles as boundaries to categorize the remaining unexplored regions. First, we divide the remaining unknown area into smaller units, such as 5*x*5 grid. Then these unknown cells are divided into two categories, one is the large undetected cells as the target cells, and the other is the cells surrounded by obstacles as the undetectable part, which are ignored.

The second stage of the AntBot-EX focuses on path planning within the known environment. Now that we have a general idea of where the unexplored regions lie (thanks to the initial exploration), we can divide these unexplored regions into different target areas. Then, we can directly employ a path-finding algorithm to identify the shortest path to each cell. Our method utilizes the A^*^ algorithm for this task. The A^*^ algorithm will calculate the shortest path length from the current location to each unexplored area.

Based on the above information, we can further derive the score formula (Eq 8 for each unknown area, and its priority.

$$\text{score}=\frac{s_{i}^{\sigma }}{d_{i}},$$ (8)

where *s*_*i*_ is the size for each unknown area, *d*_*i*_ is the shortest distance from the current position to this unknown area, and σis the parameter signed to each unexplored area. In real-life situations, the value σ could be adjusted based on the importance of different area on the map. The pseudo-code is shown in Algorithm 2, and the searching steps are shown in Fig 4(*c*) and 4(*d*), which yields more efficient routes for broader exploration. From the figure, the path planning algorithm incorporating DESS into EX-ACO-ESC outperforms the EX-ACO-ESC algorithm alone. The shorter and more direct paths generated by the combined algorithm demonstrate its superior efficiency in navigating the given complex environment with different ending destinations.

**Algorithm 2. Directed Exploration and Secondary Search (DESS).**

1: **for** each area in unexplored areas **do**

2:   Calculate area size size ← area.size

3: **end for**

4: **for** each cell in area.cells **do**

5:   Calculate distance

6:   distance ← Astar((*x*_current_,*y*_current_),cell.position , Map)

7:   Calculate score $\text{score}=\frac{s_{i}^{\sigma }}{d_{i}}$

8:   Add score and position to list

9: **end for**

10: **if** score list all_scores is not empty **then**

11:   Find best score best_score ←max (all_scores )

12:   Update target coordinates(*x*_target_,*y*_target_ )←

  best_score.position

13: **else**

14:   No valid target found target ←(0,0)

15: **end if**

16: Calculate path

17: path ← AntColony ((*x*_current_,*y*_current_),(*x*_target_,*y*_target_ ),Map)**return**

  (*x*_target_,*y*_target_ )

#### Stage 3: Boundary-aware path optimization (BAPO).

Thus far, we propose novel stage-1 and stage-2 algorithm that leverages the strengths of both the ACO and A^*^ algorithms, deriving the algorithms for the EX-ACO-ESC and DESS. By initially employing EX-ACO-ESC, the method efficiently gathers information about an unknown complex environment. Subsequently, the remaining unexplored areas are segmented, allowing for targeted path planning via the proposed DESS. While this approach guarantees full coverage, it suffers computational limitations as the search area expands. As the required coverage area increases, many areas that not containing information will also be included in the exploration, resulting in a significant increase in path length, which is especially obvious in high-complexity environments. To address this drawback, we introduce a boundary restriction mechanism within the full coverage framework, termed Boundary-Aware Path Optimization (BAPO). This mechanism optimizes the final path by ignoring low-value regions to save time and computational resources.

**Fig 5:**
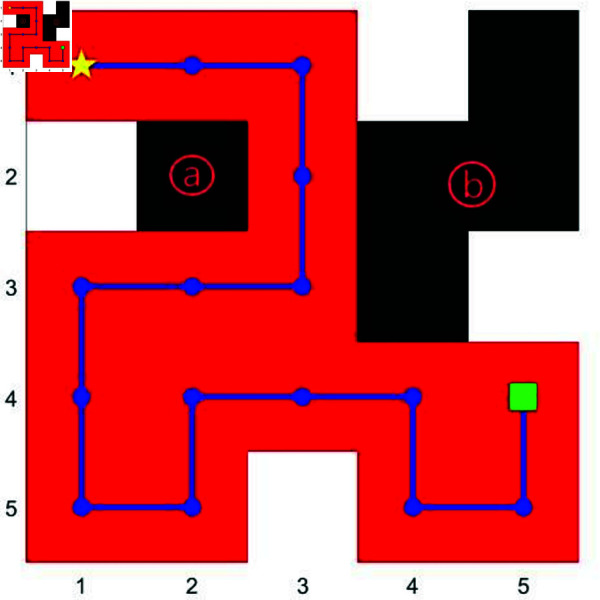
The path planning by BAPO with a configurable exploration area limit. In this figure, we establish a minimum exploration area limit of 2 grid cells for unexplored regions. The path is directed towards area *b*, designated as the target area, while area *a* is disregarded due to its size falling below the specified limit.

Our algorithm incorporates a configurable exploration area limit for the search area, allowing for flexible adaptation based on specific needs. Additionally, a mechanism is implemented to address small, potentially insignificant unexplored areas during the second stage. In the program, we will first set the coverage lower limit for the search area, such as 90%, ensuring we can collect enough information. At the same time, we set a exploration area limit for the unexplored cells during the secondary search so that regions containing low information are disregarded, as cell *a* shown in Fig 5. In practical applications, the distinction between unexplored areas will no longer be based solely on size but on score thresholds defined in Eq 8 (refer to Algorithm 3 for the pseudo-code).

**Algorithm 3. Boundary-Aware Path Optimization (BAPO).**

1: Set exploration_area_limit to *a*

2: Set target_area_limit to *b*

3: Initialize explored_area to 0

4: Initialize path_map to an empty list

5: **while** True **do**

6:   **if** explored_area > exploration_area_limit **then**

7:    Output explored_area, path_map

8:    BREAK

9:   **end if**

10:   next_area ←explore_next_area()

11:   **if** next_area < *b*

12:    CONTINUE

13:   **end if**

14:   explored_area += next_area

15:   path_map.append(next_area)

16:   **if** all_unexplored_areas_less_than(b) **then**

17:    Output explored_area, path_map

18:    BREAK

19:   **end if**

20: **end while**

## Simulation results and discussions

This study utilizes MATLAB for all simulations. The experiment involves creating random maps with adjustable obstacle densities. We systematically evaluated various algorithms by measuring their average coverage area and path length across different scenarios on these unknown maps. The results are presented in tables and figures to facilitate a comparative analysis of the algorithms’ strengths and weaknesses. To mitigate the influence of randomness, each algorithm was executed 500 times on the same random map. Given the limited existing research in this area, our data collection primarily focuses on a step-by-step comparison between the algorithms.

### A comparitive analysis of ACO-CPP and EX-ACO-ESC

**Fig 6:**
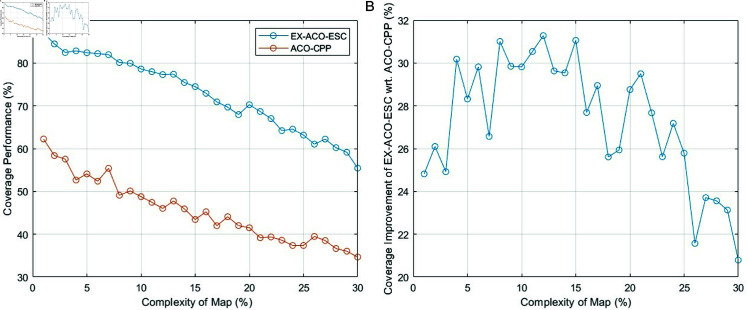
Comparison between ACO-CPP with EX-ACO-ESC in terms of coverage performance for different map complexity. (*a*): The coverage performance achieved by ACO-CPP and EX-ACO-ESC. (*b*): Coverage improvement of EX-ACO-ESC wrt. ACO-CPP

The proposed complete coverage algorithm leverages the ACO algorithm as its foundation. A key advantage of ACO in this context, compared to alternative complete coverage methods, lies in its ability to inherently avoid revisiting previously explored paths during the initial exploration phase – assuming proper configuration. This characteristic enables ACO to generate paths that are demonstrably optimal in terms of coverage for the current search area. Fig 6 is the compare between the ACO-CPP with the proposed EX-ACO-ESC.

Fig 6(a) is evident that EX-ACO-ESC maintains a significantly higher coverage across the entire range of complexities compared to ACO-CPP. As map complexity increases, both methods experience a decline in coverage, but EX-ACO-ESC’s decline is less steep, showing more stable performance. For low-complexity maps (0-10%), EX-ACO-ESC achieves nearly 90% coverage, while ACO-CPP only reaches about 60% In high-complexity maps (above 25%), EX-ACO-ESC maintains coverage above 50%, whereas ACO-CPP drops to nearly 30%. From Fig 6(b), the comparative analysis reveals that our algorithm exhibits superior performance compared to the traditional ACO-CPP. While performance fluctuations exist, performance gap is maximized between 10% −20%. Fig 6(b) also highlights certain limitations of the EX-ACO-ESC algorithm. Specifically, as map complexity surpasses 20%, the performance gap between our algorithm and the traditional ant colony algorithm diminishes. This is attributed to the algorithm’s strategy of avoiding redundant paths, which inadvertently reduces the exploration probability of unexplored regions as obstacles increase. Consequently, coverage performance deteriorates. To address this issue, further algorithmic enhancements are required to expand coverage.

### Comparitive analysis of distance-based search, unknown-size-based search and DESS

In complex environments, the primary limitation of the algorithm’s coverage area arises from its exclusive focus on maximizing coverage while strictly avoiding path redundancy. As illustrated in Fig 6, the EX-ACO-ESC algorithm demonstrates a commendable coverage rate in environments with sparse obstacles, effectively fulfilling the requirement for efficient exploration by eliminating redundant paths. However, a substantial decrease in coverage is observed with increasing obstacle density. To address this challenge, we propose incorporating a previously developed track-back mechanism to enhance coverage. This section presents a comparative analysis of multiple approaches to this problem, ultimately identifying the optimal solution.

To enhance coverage efficacy, the proposed DESS algorithm is implemented and subjected to comparative analysis alongside the distance-based search and the unknown-size-based search. The distance-based search considers the unexplored area closest to the current endpoint as the optimal exploration area. Meanwhile, the unknown-size-based search treats the largest unknown areas as the highest priority. To ensure the fairness of the experiment, all search methods will be compared under the same environment. At the same time, we will also set an exploration area limit on the exploration area for these exploration methods to observe their performance, as mentioned in BAPO. Table 1 shows the result under the complexity of 20%, Table 2 shows the result under the complexity of 30%, and Table 3 shows the result under the complexity of 40%.

**Table 1:** Comparative Analysis of three searching methods under the complexity of 20%.

	Distance-based Search		Unknown-size-based Search		DESS	
**Exploration area limit**	**Coverage**	**Path length**	**Coverage**	**Path length**	**Coverage**	**Path length**
60	71.32%	59.14	71.38%	58.52	71.17%	58.36
70	75.26%	66.61	75.74%	65.62	75.96%	65.55
80	83.15%	84.54	83.38%	80.57	83.29%	78.96
85	87.85%	95.77	87.45%	91.95	87.36%	88.74
90	92.18%	113.74	91.06%	106.37	91.45%	103.73
95	96.15%	125.82	95.46%	128.13	95.29%	121.01

**Table 2:** Comparative Analysis of three searching methods under the complexity of 30%.

	Distance-based Search		Unknown-size-based Search		DESS	
**Exploration area limit**	**Coverage**	**Path length**	**Coverage**	**Path length**	**Coverage**	**Path length**
60	66.66%	55.10	68.12%	54.58	67.26%	53.87
70	74.40%	70.42	75.41%	67.94	75.39%	66.64
80	83.24%	93.26	82.94%	84.77	83.04%	83.11
85	87.79%	108.67	87.23%	100.05	87.10%	95.43
90	91.96%	119.18	91.30%	116.97	91.25%	113.56
95	96.67%	136.52	96.12%	144.38	96.02%	130.84

**Table 3:** Comparative Analysis of three searching methods under the complexity of 40%.

	Distance-based Search		Unknown-size-based Search		DESS	
**Exploration area limit**	**Coverage**	**Path length**	**Coverage**	**Path length**	**Coverage**	**Path length**
60	63.97%	55.39	66.49%	53.78	66.30%	53.10
70	73.89%	76.20	74.18%	74.11	74.07%	71.29
80	83.29%	98.62	82.64%	91.41	82.25%	88.56
85	85.18%	105.59	85.13%	104.95	86.24%	101.18
90	92.05%	126.59	91.48%	123.16	91.28%	120.25
95	96.20%	148.32	95.76%	150.47	95.61%	142.79

Generally, as environmental complexity escalates, all methods exhibit a decline in coverage while simultaneously experiencing increased path lengths. This trend aligns with expectations, as more intricate environments pose greater challenges for exploration. A notable observation is the potential trade-off between coverage and path length among the methods. Some prioritize extensive coverage, resulting in longer paths, while others opt for shorter routes at the expense of reduced coverage. DESS demonstrates competitive performance across both metrics, suggesting its efficacy in balancing these factors.

**Fig 7:**
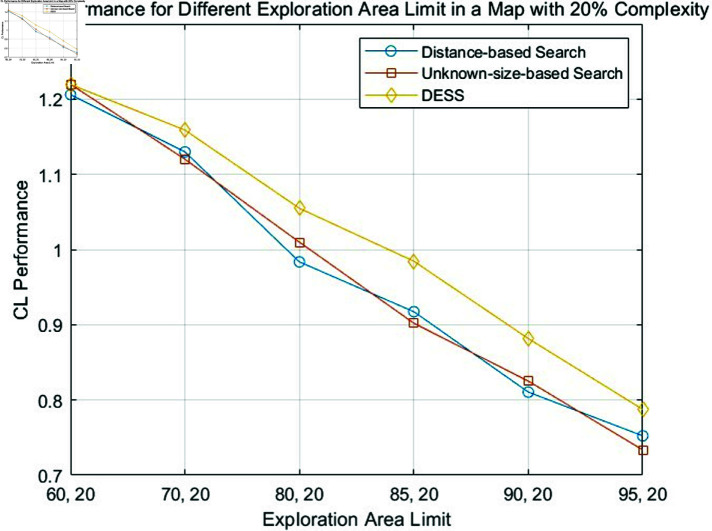
Comparison of CL performance of the distance-based search, unknown-size-based search and DESS for different exploration area limits in a fixed map complexity of 20%.

**Fig 8:**
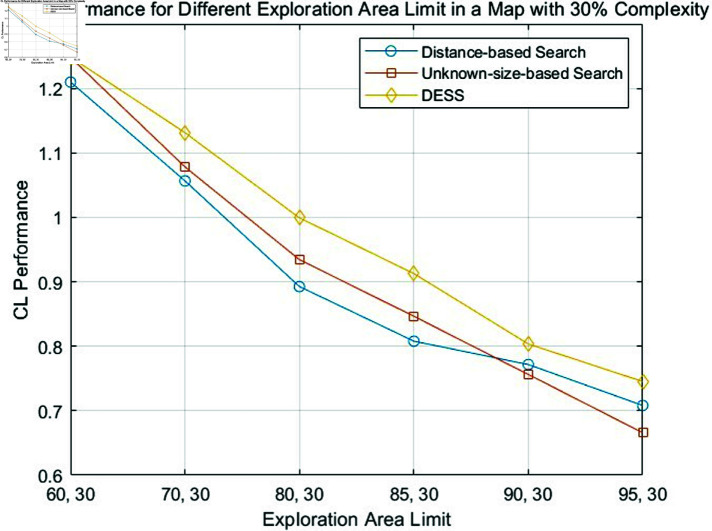
Comparison of CL performance of the distance-based search, unknown-size-based search and DESS for different exploration area limits in a fixed map complexity of 30%.

**Fig 9:**
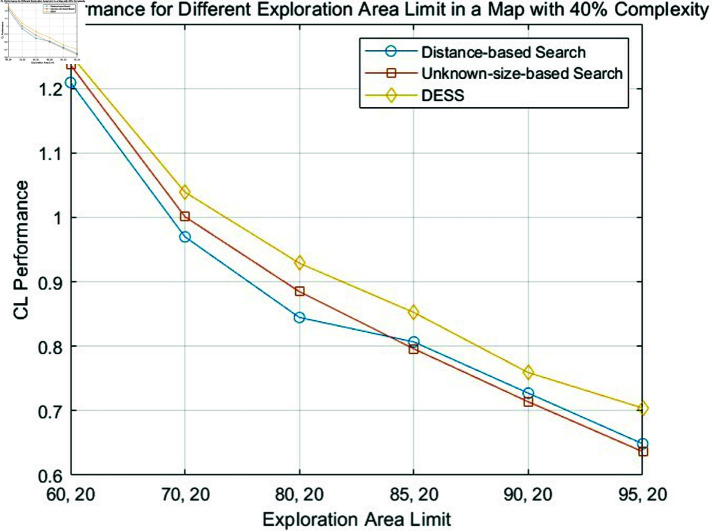
Comparison of CL performance of the distance-based search, unknown-size-based search and DESS for different exploration area limits in a fixed map complexity of 40%.

The fundamental principle of our methodology involves identifying the shortest path that maximizes the explored area within an unknown map. Eq 9 is also employed to assess each approach’s relative merits. Specifically, Eq 9 is proposed to quantify coverage efficiency relative to path length. The resulting comparative analysis is graphically depicted in Figs 7, 8, and 9.

$$CL=\frac{\text{coverage}}{\text{path length}},$$ (9)

where *CL* represents the coverage rate to path length ratio, the reduction of *CL* means that the more steps it takes to explore a unit coverage area, the lower its efficiency will be.

From Tables 1, 2, and 3 and Figs 7, 8, and 9, a general trend emerges: the search efficiency decreases with coverage lower limit increases, a predictable outcome given the challenges associated with navigating complex environments. The proposed DESS method consistently outperforms the others regarding path length across all different coverage lower limits. This method exhibits exceptional coverage and path length performance, particularly in high-complexity scenarios. Its ability to efficiently cover the environment while minimizing the distance traveled is evident. In contrast, the distance-based method basically incurs the most extended path lengths, resulting in lower efficiency. However, with the increase of coverage lower limit, its performance shows an upward trend because if all unknown areas are explored equally, it is evident that the nearest search principle will be optimal. The unknown-size search method presents a middle ground between the DESS and distance-based approaches, with moderate coverage and path length performance. A clear trade-off between coverage and path length is observed for some methods.

Meanwhile, with the comparison between different tables, we can find out that as the complexity of the map (%) increases, the path length generally increases for all methods. This suggests that navigating more complex environments becomes less efficient for all approaches. Furthermore, the figures above reveal a favorable coverage rate to path length ratio (CL), especially in challenging obstacle scenarios. These findings collectively highlight the method’s ability to effectively balance path optimization and coverage rate.

### Comparative analysis of boundary-less path optimization (BLPO) and BAPO

This section presents a comparative analysis of Boundary-Less Path Optimization (BLPO) and the proposed BAPO. These methods differ in their approach to coverage and search target, with BLPO operating without predefined exploration area limit and BAPO incorporating the pre-determined exploration area limit mentioned in the algorithm part. From Figs 7, 8, and 9, it is evident that the search efficiency will decrease as the coverage lower limit increases. This trend indicates the importance of the coverage lower limit.

Table 4 presents a comparative analysis of BLPO and BAPO algorithms across varying environmental complexities. The table evaluates the performance of these algorithms based on coverage percentage and path length. BLPO consistently achieves perfect coverage (100%) in all tested environments, demonstrating its effectiveness in exploring the entire area. However, this thoroughness comes at the cost of longer path lengths, particularly in more complex scenarios. In contrast, BAPO offers a balance between coverage and efficiency. While it doesn’t match BLPO’s perfect coverage in the most complex environments, it still achieves very high coverage rates while maintaining significantly shorter path lengths. BAPO’s performance highlights its potential for practical applications where both comprehensive coverage and optimized path planning are essential. Its ability to efficiently explore the environment while minimizing travel distance makes it a valuable option for various scenarios.

**Table 4:** Compare between BLPO and BAPO.

	BAPO		BLPO	
**Complexity**	**Coverage**	**Path length**	**Coverage**	**Path length**
3%	99.94%	135.57	100.00%	142.47
6%	99.64%	183.65	100.00%	211.67
9%	99.30%	202.17	100.00%	237.78
12%	98.82%	204.72	100.00%	264.81
15%	98.66%	206.31	99.96%	266.23
18%	96.84%	208.93	99.93%	270.02

By implementing a predefined exploration area limit, the algorithm strategically prioritizes larger regions for exploration, as these areas are more likely to yield valuable search results. Consequently, while the BAPO method sacrifices exhaustive coverage for efficiency, this trade-off is mitigated by the fact that smaller, less informative areas are excluded from the search process. This optimization results in a substantial reduction in path length without significantly compromising the overall quality of search information.

Additionally, a comparative analysis of the two methods is presented through mathematical equations below and Table 5.

**Table 5:** Further compare between BLPO and BAPO.

Complexity	Increase of path length	Increase of coverage	L	C
3%	6.90	0.06	5.09%	-0.06%
6%	28.02	0.36	15.26%	-0.36%
9%	35.61	0.70	17.62%	-0.70%
12%	60.09	1.18	29.34%	-1.19%
15%	59.92	1.30	29.04%	-1.32%
18%	61.09	3.09	29.23%	-3.19%

$$L=\frac{\Delta \text{path length}}{{\text{path length}}_{\text{with limit}}}\times 100\%,$$ (10)

$$C=\frac{\Delta \text{coverage}}{{\text{coverage}}_{\text{with limit}}}\times 100\%,$$ (11)

where Δ path length is the path length difference between the two methods, *L* and *C* are the path length and coverage improvements of the BLPO.

Table 5 gives a further analysis of the two methods. From the above calculation results, we can find out that as the map complexity increases, the coverage gap between BAPO and BLPO shows some increase. However, the decrease is minimal, with a maximum decrease of only 3.19. In contrast, the increase in path length is very significant, with the minimum percentage growth in path length being 5.09% and the maximum reaching 29.34%. This means that although the BLPO improves the coverage rate, the significant increase in path length deteriorates the overall effect. From a practical application perspective, excessive path length leads to increased energy and time consumption, resulting in higher costs. Therefore, despite the slight improvement in coverage rate, the significant increase in path length means that the unlimited method’s overall performance is declining, making it a case of diminishing returns. From a practical application perspective, we aim to address full coverage path planning on unknown maps in complex post-disaster environments. Therefore, improving efficiency and reducing time are our primary objectives, while having a small unknown area is acceptable for us. Although the unlimited method can achieve a perfect 100% coverage rate, the significant time and efficiency losses it incurs are unacceptable for our purposes.

### Comparison between ACO-CPP and Antbot-EX-CPP

Table 6 is the final comparison between AC0-CPP and Antbot-EX-CPP. In this comparison, we gradually expand the size of the grid map based on the complexity of 40% to analyze the final performance of our proposed method. It is not difficult to see from the table that when the complexity of the map reaches 40%, AC0-CPP can hardly meet the requirements, but the scalability of Antbot-EX CPP is quite good. Although the map area has been expanded, our method still performs well in the coverage area. As for the completion time, we can see that our method can complete the search easily when the map is small, but with the expansion of the map area, the computational complexity of our method, especially the time to complete the DESS part, also surges with the increase of the map area. It can be seen that our Antbot-EX method is suitable for full coverage path planning in small spaces. In large complex environments, we may still need to combine other means to simplify the calculation to shorten the analysis time.

**Table 6:** Comparison of methods based on coverage, path length, and running time for different map sizes.

Map size	Method	Coverage	Path length	Running time
Complexity (40%) limit (90%)				
20*20	ACO CPP	4.96%	10.07	0.23s
	Antbot-EX	90.08%	428	2.5s
40*40	ACO CPP	1.41%	11.48	1.31s
	Antbot-EX	90.07%	1661	15.1s
70*70	ACO CPP	1.03%	34.93	7.41s
	Antbot-EX	90.06%	5300	230.2s

## Conclusion

This paper presents a comprehensive investigation into efficient path planning strategies for achieving complete coverage in the complex and dynamic post-disaster environment. To address this challenge, we propose the AntBox-Ex framework, a three-stage approach integrating EX-ACO-ESC, DESS, and BAPO modules. Experimental results consistently demonstrate the superior performance of our framework in terms of coverage rate and path length, especially in obstacle-rich terrains. By effectively balancing exploration and exploitation, the AntBox-Ex framework offers a significant advancement in robotic navigation and coverage. These findings hold immense potential for enhancing the efficiency and effectiveness of autonomous search and rescue operations. However, combined with the high coverage percentage, there are also some limitations associated with our method. The first is that our method might suffer a high running time with increasing map. Meanwhile, real-world deployment poses additional challenges, including sensor inaccuracies, hardware constraints, and environmental unpredictability. To bridge this gap, future work will focus on reducing DESS complexity, such as more rational allocation of the grid size or combining with more advanced methods to shorten running time. Furthermore, we will also try to test our AntBot-EX method on physical robots in controlled environments with simulated debris and dynamic obstacles.
