# Supplementary material for: AntBot-EX: Enhancing robot search efficiency in complex post-disaster environments
Source: PLoS One. 2025 May 22;20(5):e0322980. doi: 10.1371/journal.pone.0322980 (PMC12097565; doi:10.1371/journal.pone.0322980)
Supplement: Attachment — Submitted filename: Response to Reviewers.docx [file pone.0322980.s001.docx]

**Manuscript ID:**

Original Article Title: AntBot-EX: Enhancing Robot Search Efficiency in Complex Post-Disaster Environments

**Amended Article Title**: AntBot-EX: Enhancing Robot Search Efficiency in Complex Post-Disaster Environments

**To:** Editor of Plos One

**Re:** Response to reviewers

Thank you for allowing a resubmission of our manuscript, with an opportunity to address the reviewers' comments. We are uploading the following three files:

(a) the response letter to the comments of reviewers;

(b) the revised paper with track changes;

(c) a clean revised paper.

Best regards,

Yao

**Reviewer 1**

**We would like to take this opportunity to thank the reviewers for their constructive comments and suggestions in improving the quality and readability of this manuscript. Your effort and time in reviewing this paper are highly appreciated. We have rigorously revised the manuscript based on the reviewers' comments as follows:**

Comment 1: The simulation results support the claims made, but additional real-world testing or case studies would strengthen the relevance and applicability of the proposed approach.

We sincerely appreciate the reviewer’s valuable suggestion to enhance the practical relevance of our work through real-world testing or case studies. While our current experiments are entirely simulation-based due to resource constraints (e.g., hardware availability and access to disaster environments), we acknowledge that real-world deployment introduces challenges such as sensor noise, dynamic obstacles, and hardware limitations.

Our simulations rigorously validated the proposed AntBot-EX framework in diverse grid-based environments with varying obstacle densities (up to 40% complexity), demonstrating its adaptability to dynamic and unknown scenarios. These results serve as a foundational proof of concept, highlighting the algorithm’s potential for real-world applications. We have added further explanation in the revised manuscript(Section: system model), and we would like to point out that through random map generation and random distribution of obstacles, our grid map could have the ability to simulate the shape and distribution of obstacles in complex real-world environments

Furthermore, to address the reviewer’s concern, we have revised the Section: Conclusion to outline plans to test AntBot-EX in controlled laboratory environments mimicking post-disaster conditions (e.g., collapsed structures, debris fields).

Amendment:

- Revised Section: System model and Section: Conclusion

- Page 5, Section: System model Page 20, Section: Conclusion

Comment 2: While the manuscript does a good job of explaining the proposed method, some of the comparisons with traditional methods could be more critical. For instance, while the AntBot-EX shows improvement in coverage and path optimization, the manuscript should explore the trade-offs more explicitly, such as potential computational overhead or limitations in scalability.

Author response: We thank the reviewer for this valuable feedback. In the revised manuscript, we have expanded the discussion in Section 4.3 to include a detailed analysis of the computational overhead and scalability of AntBot-EX compared to traditional methods. We have also added a table (Table 6) summarizing the trade-offs between map size, coverage, path length, and computational complexity.

Amendment:

- Expanded Comparative Analysis of Computational Overhead and Scalability

- Added Table 6: Comparison of methods based on coverage, path length, and running time for different map sizes.

- Page 19, Section: Comparative Analysis of Computational Overhead and Scalability

Comment 3: Consider including a flowchart or diagram to visually represent the stages of the AntBot-EX framework, which would aid in understanding the methodology.

Author response: We agree with the reviewer that a visual representation of the AntBot-EX framework would enhance the clarity of the methodology. In response, we have added a new figure (Figure 2) that illustrates the three-stage workflow of AntBot-EX, including the EX-ACO-ESC, DESS, and BAPO stages.

Amendment:

- Added Figure 2: Flowchart of the AntBot-EX Framework

- Page 6, Section: System model and Algorithms

Comment 4: Some sections, particularly the methodology, are dense with technical details, which might be overwhelming for some readers. Consider simplifying the language or breaking down complex sentences to enhance readability.

Author response: We appreciate the reviewer's suggestion to improve the readability of the methodology section. In the revised manuscript, we have simplified the language in Section 3 and broken down complex sentences into more digestible parts. Additionally, we have added more explanatory text to help readers understand the technical details.

Amendment:

- Simplified language in Section: Methodology

- Added explanatory text to clarify technical details

- Pages 5-12, Section: Methodology

**End**

**Reviewer 2**

**We would like to take this opportunity to thank the reviewers for their constructive comments and suggestions in improving the quality and readability of this manuscript. Your effort and time in reviewing this paper are highly appreciated. We have rigorously revised the manuscript based on the reviewers' comments as follows:**

Comment 1: The focus on SAR in disaster zones is timely and impactful, with direct implications for saving lives.

Author response: Thank you for your comment, we will make more efforts in this direction in the future.

Comment 2: The integration of the modified ACO with escape mechanisms, directed exploration, and boundary-aware optimization demonstrates a novel and well-structured approach to addressing CPP challenges.

Author response: Thank you for your approval of our approach.

Comment 3: Clarity of Writing: The paper is well-structured, with clear articulation of the problem, methodology, and findings.

Author response: Thank you for your comment.

Comment 4: The simulations are robust, but real-world deployment scenarios are absent. The results may differ in real disaster environments due to hardware limitations, sensor inaccuracies, or environmental unpredictability.

We sincerely appreciate the reviewer’s valuable suggestion to enhance the practical relevance of our work through real-world testing or case studies. While our current experiments are entirely simulation-based due to resource constraints (e.g., hardware availability and access to disaster environments), we acknowledge that real-world deployment introduces challenges such as sensor noise, dynamic obstacles, and hardware limitations.

Our simulations rigorously validated the proposed AntBot-EX framework in diverse grid-based environments with varying obstacle densities (up to 40% complexity), demonstrating its adaptability to dynamic and unknown scenarios. These results serve as a foundational proof of concept, highlighting the algorithm’s potential for real-world applications. We have added further explanation in the revised manuscript(Section: system model), and we would like to point out that through random map generation and random distribution of obstacles, our grid map could have the ability to simulate the shape and distribution of obstacles in complex real-world environments

Furthermore, to address the reviewer’s concern, we have revised the Section: Conclusion to outline plans to test AntBot-EX in controlled laboratory environments mimicking post-disaster conditions (e.g., collapsed structures, debris fields).

Amendment:

- revised Section: System model and Section: Conclusion

- Page 5, Section: System model Page 20, Section: Conclusion

Comment 5: While computational efficiency is critical, the paper could provide a deeper discussion on the practical challenges of implementation, such as computational overhead, power consumption, and real-time decision-making.

Author response: We thank the reviewer for this suggestion. In the revised manuscript, we have expanded the discussion in Section: Comparative Analysis of Computational Overhead and Scalability, to include a detailed analysis of computational overhead, power consumption, and real-time decision-making challenges. We have also added a table (Table 6) summarizing these practical challenges.

Amendment:

- Expanded Section : Comparative Analysis of Computational Overhead and Scalability

- Added Table 6: Comparison of methods based on coverage, path length, and running time for different map sizes.

- Page 19, Section: Comparative Analysis of Computational Overhead and Scalability

Comment 6: Although comparisons with traditional methods (e.g., ACO-CPP and DESS) are detailed, additional comparisons with state-of-the-art machine learning-based or hybrid CPP methods could strengthen the study.

Author response: We appreciate the reviewer's suggestion to include comparisons with state-of-the-art machine learning-based or hybrid CPP methods. At present, the use of reinforcement learning and machine learning for full coverage path planning has indeed become the main research direction, and we have also begun to carry out further simulation of full coverage path planning combined with reinforcement learning. In the next article, we will make a detailed comparison between our Antbot-EX method and the reinforcement learning method.

**End**
