# Supplementary material for: AntBot-EX: Enhancing robot search efficiency in complex post-disaster environments
Source: PLoS One. 2025 May 22;20(5):e0322980. doi: 10.1371/journal.pone.0322980 (PMC12097565; doi:10.1371/journal.pone.0322980)
Supplement: Peer review history

# Author comment: Author response to Decision Letter 0

**DOI:** 10.1371/journal.pone.0322980.r001

2 Aug 2024

# Peer review: Decision Letter 0

**DOI:** 10.1371/journal.pone.0322980.r002

**Authors:** Hashim Ali

21 Jan 2025

PONE-D-24-32527AntBot-EX: Enhancing Robot Search Efficiency in Complex Post-Disaster EnvironmentsPLOS ONE

Dear Dr. Xue,

Thank you for submitting your manuscript to PLOS ONE. After careful consideration, we feel that it has merit but does not fully meet PLOS ONE’s publication criteria as it currently stands. Therefore, we invite you to submit a revised version of the manuscript that addresses the points raised during the review process. **We appreciate your patience during the review period. I would like to personally apologize for the delay in the review process, which was unusually lengthy in this case. We experienced difficulty in finding relevant experts to review your manuscript, which contributed significantly to the delay. We are committed to ensuring a thorough and fair review process and are taking steps to prevent such delays in the future. Thank you for your understanding.**

Please submit your revised manuscript by Mar 07 2025 11:59PM. If you will need more time than this to complete your revisions, please reply to this message or contact the journal office at plosone@plos.org. When you're ready to submit your revision, log on to https://www.editorialmanager.com/pone/ and select the 'Submissions Needing Revision' folder to locate your manuscript file.

Please include the following items when submitting your revised manuscript:

- A rebuttal letter that responds to each point raised by the academic editor and reviewer(s). You should upload this letter as a separate file labeled 'Response to Reviewers'.
- A marked-up copy of your manuscript that highlights changes made to the original version. You should upload this as a separate file labeled 'Revised Manuscript with Track Changes'.
- An unmarked version of your revised paper without tracked changes. You should upload this as a separate file labeled 'Manuscript'.

If you would like to make changes to your financial disclosure, please include your updated statement in your cover letter. Guidelines for resubmitting your figure files are available below the reviewer comments at the end of this letter.

If applicable, we recommend that you deposit your laboratory protocols in protocols.io to enhance the reproducibility of your results. Protocols.io assigns your protocol its own identifier (DOI) so that it can be cited independently in the future. For instructions see: https://journals.plos.org/plosone/s/submission-guidelines#loc-laboratory-protocols. Additionally, PLOS ONE offers an option for publishing peer-reviewed Lab Protocol articles, which describe protocols hosted on protocols.io. Read more information on sharing protocols at https://plos.org/protocols?utm_medium=editorial-email&utm_source=authorletters&utm_campaign=protocols.

We look forward to receiving your revised manuscript.

Kind regards,

Hashim Ali

Academic Editor

PLOS ONE

**Journal Requirements:**

1. When submitting your revision, we need you to address these additional requirements.

Please ensure that your manuscript meets PLOS ONE's style requirements, including those for file naming. The PLOS ONE style templates can be found at 

https://journals.plos.org/plosone/s/file?id=wjVg/PLOSOne_formatting_sample_main_body.pdf and 

https://journals.plos.org/plosone/s/file?id=ba62/PLOSOne_formatting_sample_title_authors_affiliations.pdf

2. Please note that PLOS ONE has specific guidelines on code sharing for submissions in which author-generated code underpins the findings in the manuscript. In these cases, all author-generated code must be made available without restrictions upon publication of the work. Please review our guidelines at https://journals.plos.org/plosone/s/materials-and-software-sharing#loc-sharing-code and ensure that your code is shared in a way that follows best practice and facilitates reproducibility and reuse.

3. We note that your Data Availability Statement is currently as follows: All relevant data are within the manuscript and its Supporting Information files.

Please confirm at this time whether or not your submission contains all raw data required to replicate the results of your study. Authors must share the “minimal data set” for their submission. PLOS defines the minimal data set to consist of the data required to replicate all study findings reported in the article, as well as related metadata and methods (https://journals.plos.org/plosone/s/data-availability#loc-minimal-data-set-definition).

For example, authors should submit the following data:

- The values behind the means, standard deviations and other measures reported;

- The values used to build graphs;

- The points extracted from images for analysis.

Authors do not need to submit their entire data set if only a portion of the data was used in the reported study.

If your submission does not contain these data, please either upload them as Supporting Information files or deposit them to a stable, public repository and provide us with the relevant URLs, DOIs, or accession numbers. For a list of recommended repositories, please see https://journals.plos.org/plosone/s/recommended-repositories.

If there are ethical or legal restrictions on sharing a de-identified data set, please explain them in detail (e.g., data contain potentially sensitive information, data are owned by a third-party organization, etc.) and who has imposed them (e.g., an ethics committee). Please also provide contact information for a data access committee, ethics committee, or other institutional body to which data requests may be sent. If data are owned by a third party, please indicate how others may request data access.

4. Please review your reference list to ensure that it is complete and correct. If you have cited papers that have been retracted, please include the rationale for doing so in the manuscript text, or remove these references and replace them with relevant current references. Any changes to the reference list should be mentioned in the rebuttal letter that accompanies your revised manuscript. If you need to cite a retracted article, indicate the article’s retracted status in the References list and also include a citation and full reference for the retraction notice.

[Note: HTML markup is below. Please do not edit.]

Reviewers' comments:

Reviewer's Responses to Questions

**Comments to the Author**

1. Is the manuscript technically sound, and do the data support the conclusions?

The manuscript must describe a technically sound piece of scientific research with data that supports the conclusions. Experiments must have been conducted rigorously, with appropriate controls, replication, and sample sizes. The conclusions must be drawn appropriately based on the data presented. 

Reviewer #1: Yes

Reviewer #2: Yes

**********

2. Has the statistical analysis been performed appropriately and rigorously? 

Reviewer #1: Yes

Reviewer #2: Yes

**********

3. Have the authors made all data underlying the findings in their manuscript fully available?

The PLOS Data policy requires authors to make all data underlying the findings described in their manuscript fully available without restriction, with rare exception (please refer to the Data Availability Statement in the manuscript PDF file). The data should be provided as part of the manuscript or its supporting information, or deposited to a public repository. For example, in addition to summary statistics, the data points behind means, medians and variance measures should be available. If there are restrictions on publicly sharing data—e.g. participant privacy or use of data from a third party—those must be specified.

Reviewer #1: Yes

Reviewer #2: Yes

**********

4. Is the manuscript presented in an intelligible fashion and written in standard English?

PLOS ONE does not copyedit accepted manuscripts, so the language in submitted articles must be clear, correct, and unambiguous. Any typographical or grammatical errors should be corrected at revision, so please note any specific errors here.

Reviewer #1: Yes

Reviewer #2: Yes

**********

5. Review Comments to the Author

Please use the space provided to explain your answers to the questions above. You may also include additional comments for the author, including concerns about dual publication, research ethics, or publication ethics. (Please upload your review as an attachment if it exceeds 20,000 characters)

**Reviewer #1:** The manuscript presents a novel approach to improving search efficiency in post-disaster environments using the AntBot-EX framework, which combines multiple algorithms to address the limitations of traditional Coverage Path Planning (CPP) methods. The content is accurate, relevant, and well-aligned with the research question. The focus on enhancing robot search efficiency in dynamic and unpredictable environments is timely and significant given the increasing reliance on autonomous systems in disaster management.

Few suggestions:

- The simulation results support the claims made, but additional real-world testing or case studies would strengthen the relevance and applicability of the proposed approach.

- While the manuscript does a good job of explaining the proposed method, some of the comparisons with traditional methods could be more critical. For instance, while the AntBot-EX shows improvement in coverage and path optimization, the manuscript should explore the trade-offs more explicitly, such as potential computational overhead or limitations in scalability.

-Consider including a flowchart or diagram to visually represent the stages of the AntBot-EX framework, which would aid in understanding the methodology.

- Some sections, particularly the methodology, are dense with technical details, which might be overwhelming for some readers. Consider simplifying the language or breaking down complex sentences to enhance readability.

**Reviewer #2: **The manuscript addresses a critical issue in robotic search and rescue (SAR) operations, particularly in post-disaster environments. The authors propose a three-stage Coverage Path Planning (CPP) method named AntBot-EX, integrating a modified Ant Colony Optimization algorithm (ACO), the A* algorithm, and a Boundary-Aware Path Optimization (BAPO) mechanism. The method aims to address challenges such as dynamic obstacles, path redundancy, and computational efficiency.

My comments point by point about the paper are:

1. The focus on SAR in disaster zones is timely and impactful, with direct implications for saving lives.

2. The integration of the modified ACO with escape mechanisms, directed exploration, and boundary-aware optimization demonstrates a novel and well-structured approach to addressing CPP challenges.

3. Clarity of Writing: The paper is well-structured, with clear articulation of the problem, methodology, and findings.

4. The simulations are robust, but real-world deployment scenarios are absent. The results may differ in real disaster environments due to hardware limitations, sensor inaccuracies, or environmental unpredictability.

5. While computational efficiency is critical, the paper could provide a deeper discussion on the practical challenges of implementation, such as computational overhead, power consumption, and real-time decision-making.

6. Although comparisons with traditional methods (e.g., ACO-CPP and DESS) are detailed, additional comparisons with state-of-the-art machine learning-based or hybrid CPP methods could strengthen the study.

The manuscript contributes significantly to the field of robotic SAR operations by addressing key CPP challenges with a structured, innovative approach. Minor revisions focusing on real-world application, parameter analysis, and broader comparisons will enhance the study’s impact.

**********

6. PLOS authors have the option to publish the peer review history of their article (what does this mean?). If published, this will include your full peer review and any attached files.

If you choose “no”, your identity will remain anonymous but your review may still be made public.

**Do you want your identity to be public for this peer review?** For information about this choice, including consent withdrawal, please see our Privacy Policy.

Reviewer #1: No

Reviewer #2: No

**********

[NOTE: If reviewer comments were submitted as an attachment file, they will be attached to this email and accessible via the submission site. Please log into your account, locate the manuscript record, and check for the action link "View Attachments". If this link does not appear, there are no attachment files.]

While revising your submission, please upload your figure files to the Preflight Analysis and Conversion Engine (PACE) digital diagnostic tool, https://pacev2.apexcovantage.com/. PACE helps ensure that figures meet PLOS requirements. To use PACE, you must first register as a user. Registration is free. Then, login and navigate to the UPLOAD tab, where you will find detailed instructions on how to use the tool. If you encounter any issues or have any questions when using PACE, please email PLOS at figures@plos.org. Please note that Supporting Information files do not need this step.

# Author comment: Author response to Decision Letter 1

**DOI:** 10.1371/journal.pone.0322980.r003

25 Feb 2025

Editor

1. The manuscript is written under PLOS ONE Latex template.

2. I have submit my code in manuscript data part.

3. All my data comes from simulations of the code I provided.

Reviewer one:

Comment 1: The simulation results support the claims made, but additional real-world testing or case studies would strengthen the relevance and applicability of the proposed approach.

We sincerely appreciate the reviewer’s valuable suggestion to enhance the practical relevance of our work through real-world testing or case studies. While our current experiments are entirely simulation-based due to resource constraints (e.g., hardware availability and access to disaster environments), we acknowledge that real-world deployment introduces challenges such as sensor noise, dynamic obstacles, and hardware limitations.

Our simulations rigorously validated the proposed AntBot-EX framework in diverse grid-based environments with varying obstacle densities (up to 40% complexity), demonstrating its adaptability to dynamic and unknown scenarios. These results serve as a foundational proof of concept, highlighting the algorithm’s potential for real-world applications. We have added further explanation in the revised manuscript(Section: system model), and we would like to point out that through random map generation and random distribution of obstacles, our grid map could have the ability to simulate the shape and distribution of obstacles in complex real-world environments

Furthermore, to address the reviewer’s concern, we have revised the Section: Conclusion to outline plans to test AntBot-EX in controlled laboratory environments mimicking post-disaster conditions (e.g., collapsed structures, debris fields).

Amendment:

- Revised Section: System model and Section: Conclusion

- Page 5, Section: System model Page 20, Section: Conclusion

Comment 2: While the manuscript does a good job of explaining the proposed method, some of the comparisons with traditional methods could be more critical. For instance, while the AntBot-EX shows improvement in coverage and path optimization, the manuscript should explore the trade-offs more explicitly, such as potential computational overhead or limitations in scalability.

Author response: We thank the reviewer for this valuable feedback. In the revised manuscript, we have expanded the discussion in Section 4.3 to include a detailed analysis of the computational overhead and scalability of AntBot-EX compared to traditional methods. We have also added a table (Table 6) summarizing the trade-offs between map size, coverage, path length, and computational complexity.

Amendment:

- Expanded Comparative Analysis of Computational Overhead and Scalability

- Added Table 6: Comparison of methods based on coverage, path length, and running time for different map sizes.

- Page 19, Section: Comparative Analysis of Computational Overhead and Scalability

Comment 3: Consider including a flowchart or diagram to visually represent the stages of the AntBot-EX framework, which would aid in understanding the methodology.

Author response: We agree with the reviewer that a visual representation of the AntBot-EX framework would enhance the clarity of the methodology. In response, we have added a new figure (Figure 2) that illustrates the three-stage workflow of AntBot-EX, including the EX-ACO-ESC, DESS, and BAPO stages.

Amendment:

- Added Figure 2: Flowchart of the AntBot-EX Framework

- Page 6, Section: System model and Algorithms

Comment 4: Some sections, particularly the methodology, are dense with technical details, which might be overwhelming for some readers. Consider simplifying the language or breaking down complex sentences to enhance readability.

Author response: We appreciate the reviewer's suggestion to improve the readability of the methodology section. In the revised manuscript, we have simplified the language in Section 3 and broken down complex sentences into more digestible parts. Additionally, we have added more explanatory text to help readers understand the technical details.

Amendment:

- Simplified language in Section: Methodology

- Added explanatory text to clarify technical details

- Pages 5-12, Section: Methodology

Reviewer two:

Comment 1: The focus on SAR in disaster zones is timely and impactful, with direct implications for saving lives.

Author response: Thank you for your comment, we will make more efforts in this direction in the future.

Comment 2: The integration of the modified ACO with escape mechanisms, directed exploration, and boundary-aware optimization demonstrates a novel and well-structured approach to addressing CPP challenges.

Author response: Thank you for your approval of our approach.

Comment 3: Clarity of Writing: The paper is well-structured, with clear articulation of the problem, methodology, and findings.

Author response: Thank you for your comment.

Comment 4: The simulations are robust, but real-world deployment scenarios are absent. The results may differ in real disaster environments due to hardware limitations, sensor inaccuracies, or environmental unpredictability.

We sincerely appreciate the reviewer’s valuable suggestion to enhance the practical relevance of our work through real-world testing or case studies. While our current experiments are entirely simulation-based due to resource constraints (e.g., hardware availability and access to disaster environments), we acknowledge that real-world deployment introduces challenges such as sensor noise, dynamic obstacles, and hardware limitations.

Our simulations rigorously validated the proposed AntBot-EX framework in diverse grid-based environments with varying obstacle densities (up to 40% complexity), demonstrating its adaptability to dynamic and unknown scenarios. These results serve as a foundational proof of concept, highlighting the algorithm’s potential for real-world applications. We have added further explanation in the revised manuscript(Section: system model), and we would like to point out that through random map generation and random distribution of obstacles, our grid map could have the ability to simulate the shape and distribution of obstacles in complex real-world environments

Furthermore, to address the reviewer’s concern, we have revised the Section: Conclusion to outline plans to test AntBot-EX in controlled laboratory environments mimicking post-disaster conditions (e.g., collapsed structures, debris fields).

Amendment:

- revised Section: System model and Section: Conclusion

- Page 5, Section: System model Page 20, Section: Conclusion

Comment 5: While computational efficiency is critical, the paper could provide a deeper discussion on the practical challenges of implementation, such as computational overhead, power consumption, and real-time decision-making.

Author response: We thank the reviewer for this suggestion. In the revised manuscript, we have expanded the discussion in Section: Comparative Analysis of Computational Overhead and Scalability, to include a detailed analysis of computational overhead, power consumption, and real-time decision-making challenges. We have also added a table (Table 6) summarizing these practical challenges.

Amendment:

- Expanded Section : Comparative Analysis of Computational Overhead and Scalability

- Added Table 6: Comparison of methods based on coverage, path length, and running time for different map sizes.

- Page 19, Section: Comparative Analysis of Computational Overhead and Scalability

Comment 6: Although comparisons with traditional methods (e.g., ACO-CPP and DESS) are detailed, additional comparisons with state-of-the-art machine learning-based or hybrid CPP methods could strengthen the study.

Author response: We appreciate the reviewer's suggestion to include comparisons with state-of-the-art machine learning-based or hybrid CPP methods. At present, the use of reinforcement learning and machine learning for full coverage path planning has indeed become the main research direction, and we have also begun to carry out further simulation of full coverage path planning combined with reinforcement learning. In the next article, we will make a detailed comparison between our Antbot-EX method and the reinforcement learning method.

Attachment Submitted filename: Response to Reviewers.docx

# Peer review: Decision Letter 1

**DOI:** 10.1371/journal.pone.0322980.r004

**Authors:** Hashim Ali

1 Apr 2025

AntBot-EX: Enhancing Robot Search Efficiency in Complex Post-Disaster Environments

PONE-D-24-32527R1

Dear Dr. Xue,

We’re pleased to inform you that your manuscript has been judged scientifically suitable for publication and will be formally accepted for publication once it meets all outstanding technical requirements.

Within one week, you’ll receive an e-mail detailing the required amendments. When these have been addressed, you’ll receive a formal acceptance letter and your manuscript will be scheduled for publication.

An invoice will be generated when your article is formally accepted. Please note, if your institution has a publishing partnership with PLOS and your article meets the relevant criteria, all or part of your publication costs will be covered. Please make sure your user information is up-to-date by logging into Editorial Manager at Editorial Manager® and clicking the ‘Update My Information' link at the top of the page. If you have any questions relating to publication charges, please contact our Author Billing department directly at authorbilling@plos.org.

If your institution or institutions have a press office, please notify them about your upcoming paper to help maximize its impact. If they’ll be preparing press materials, please inform our press team as soon as possible -- no later than 48 hours after receiving the formal acceptance. Your manuscript will remain under strict press embargo until 2 pm Eastern Time on the date of publication. For more information, please contact onepress@plos.org.

Kind regards,

Hashim Ali, Ph.D.

Academic Editor

PLOS ONE

Additional Editor Comments (optional):

Reviewers' comments:

Reviewer's Responses to Questions

**Comments to the Author**

1. If the authors have adequately addressed your comments raised in a previous round of review and you feel that this manuscript is now acceptable for publication, you may indicate that here to bypass the “Comments to the Author” section, enter your conflict of interest statement in the “Confidential to Editor” section, and submit your "Accept" recommendation.

Reviewer #1: All comments have been addressed

Reviewer #2: All comments have been addressed

**********

2. Is the manuscript technically sound, and do the data support the conclusions?

The manuscript must describe a technically sound piece of scientific research with data that supports the conclusions. Experiments must have been conducted rigorously, with appropriate controls, replication, and sample sizes. The conclusions must be drawn appropriately based on the data presented. 

Reviewer #1: Yes

Reviewer #2: Yes

**********

3. Has the statistical analysis been performed appropriately and rigorously? 

Reviewer #1: Yes

Reviewer #2: Yes

**********

4. Have the authors made all data underlying the findings in their manuscript fully available?

The PLOS Data policy requires authors to make all data underlying the findings described in their manuscript fully available without restriction, with rare exception (please refer to the Data Availability Statement in the manuscript PDF file). The data should be provided as part of the manuscript or its supporting information, or deposited to a public repository. For example, in addition to summary statistics, the data points behind means, medians and variance measures should be available. If there are restrictions on publicly sharing data—e.g. participant privacy or use of data from a third party—those must be specified.

Reviewer #1: Yes

Reviewer #2: Yes

**********

5. Is the manuscript presented in an intelligible fashion and written in standard English?

PLOS ONE does not copyedit accepted manuscripts, so the language in submitted articles must be clear, correct, and unambiguous. Any typographical or grammatical errors should be corrected at revision, so please note any specific errors here.

Reviewer #1: Yes

Reviewer #2: Yes

**********

6. Review Comments to the Author

Please use the space provided to explain your answers to the questions above. You may also include additional comments for the author, including concerns about dual publication, research ethics, or publication ethics. (Please upload your review as an attachment if it exceeds 20,000 characters)

Reviewer #1: The authors have made substantial improvements in response to previous feedback, addressing many of the key concerns. The article can be accepted.

Reviewer #2: (No Response)

**********

7. PLOS authors have the option to publish the peer review history of their article (what does this mean?). If published, this will include your full peer review and any attached files.

If you choose “no”, your identity will remain anonymous but your review may still be made public.

**Do you want your identity to be public for this peer review?** For information about this choice, including consent withdrawal, please see our Privacy Policy.

Reviewer #1: No

Reviewer #2: No

**********

# Editor report: Acceptance letter

**DOI:** 10.1371/journal.pone.0322980.r005

**Authors:** Hashim Ali

PONE-D-24-32527R1

PLOS ONE

Dear Dr. Xue,

I'm pleased to inform you that your manuscript has been deemed suitable for publication in PLOS ONE. Congratulations! Your manuscript is now being handed over to our production team.

At this stage, our production department will prepare your paper for publication. This includes ensuring the following:

* All references, tables, and figures are properly cited

* All relevant supporting information is included in the manuscript submission,

* There are no issues that prevent the paper from being properly typeset

If revisions are needed, the production department will contact you directly to resolve them. If no revisions are needed, you will receive an email when the publication date has been set. At this time, we do not offer pre-publication proofs to authors during production of the accepted work. Please keep in mind that we are working through a large volume of accepted articles, so please give us a few weeks to review your paper and let you know the next and final steps.

Lastly, if your institution or institutions have a press office, please let them know about your upcoming paper now to help maximize its impact. If they'll be preparing press materials, please inform our press team within the next 48 hours. Your manuscript will remain under strict press embargo until 2 pm Eastern Time on the date of publication. For more information, please contact onepress@plos.org.

If we can help with anything else, please email us at customercare@plos.org.

Thank you for submitting your work to PLOS ONE and supporting open access.

Kind regards,

PLOS ONE Editorial Office Staff

on behalf of

Dr. Hashim Ali

Academic Editor

PLOS ONE
